# Comparison of Liver Detargeting Strategies for Systemic Therapy with Oncolytic Adenovirus Serotype 5

**DOI:** 10.3390/biomedicines5030046

**Published:** 2017-08-10

**Authors:** Tien V. Nguyen, Mary E. Barry, Mallory A. Turner, Catherine M. Crosby, Miguel A. Trujillo, John C. Morris, Michael A. Barry

**Affiliations:** 1 Department of Internal Medicine, Division of Infectious Diseases, Translational Immunovirology and Biodefense Program, Mayo Clinic, Rochester, MN 55902, USA; nguyen.tien@mayo.edu (T.V.N.); mbarry@mayo.edu (M.E.B.); 2 Virology and Gene Therapy Graduate Program, Mayo Clinic, Rochester, MN 55902, USA; mallory.a.turner@gmail.com (M.A.T.); ccrosby@lji.org (C.M.C.); 3 Department of Endocrinology, Mayo Clinic, Rochester, MN 55902, USA; rtrujillo.miguel@mayo.edu (M.A.T.); morris.john@mayo.edu (J.C.M.); 4 Department of Molecular Medicine, Mayo Clinic, Rochester, MN 55902, USA; 5 Department of Immunology, Mayo Clinic, Rochester, MN 55902, USA

**Keywords:** adenovirus, conditionally-replicating adenovirus, hexon, PEG

## Abstract

Oncolytic viruses would ideally be of use for systemic therapy to treat disseminated cancer. To do this safely, this may require multiple layers of cancer specificity. The pharmacology and specificity of oncolytic adenoviruses can be modified by (1) physical retargeting, (2) physical detargeting, (3) chemical shielding, or (4) by modifying the ability of viral early gene products to selectively activate in cancer versus normal cells. We explored the utility of these approaches with oncolytic adenovirus serotype 5 (Ad5) in immunocompetent Syrian hamsters bearing subcutaneous HaK tumors. After a single intravenous injection to reach the distant tumors, the physically hepatocyte-detargeted virus Ad5-hexon-BAP was more effective than conditionally replicating Ad5-*dl*1101/07 with mutations in its E1A protein. When these control or Ad5 treated animals were treated a second time by intratumoral injection, prior exposure to Ad5 did not affect tumor growth, suggesting that anti-Ad immunity neither prevented treatment nor amplified anti-tumor immune responses. Ad5-*dl*1101/07 was next chemically shielded with polyethylene glycol (PEG). While 5 kDa of PEG blunted pro-inflammatory IL-6 production induced by Ad5-*dl*1101/07, this shielding reduced Ad oncolytic activity.

## 1. Introduction

Adenoviruses (Ads) are non-enveloped double-stranded DNA viruses that are being tested for oncolytic virotherapy (reviewed in [1,2]). These self-amplifying anticancer agents may be useful as systemic therapies against metastatic cancer. However, after intravenous (i.v.) injection, the vast majority of systemically injected adenovirus serotype 5 (Ad5) vectors are phagocytosed and destroyed by liver Kupffer cell macrophages [3,4,5]. Ad5 virions that escape Kupffer cells can be pinocytosed by liver sinusoidal endothelial cells for destruction or off-target cell infection [6]. If Ad5 evades Kupffer and endothelial cells, the virus efficiently infects hepatocytes in the parenchyma of the liver (reviewed in [7]). This effect appears to be mediated by the binding of vitamin K-dependent clotting factors including protein C, FVII, FIX, and FX to the hexon protein of Ad5 [8,9,10,11]. 

If Ad5 is fully replication-competent, the virus can cause dose-limiting hepatotoxicity or death [12,13]. Ad5 must evade these pharmacological dead ends and these sources of side effects before it can even reach distant tumors for systemic therapy [7]. Potential approaches include retargeting or detargeting the virus to control which cells are infected. Once infection has occurred, one can aim to suppress the virus’ killing of off-target cells by re-engineering the early genes of Ad to make it a conditionally-replicating Ad (CRAd) [7].

We explored the role of Kupffer cells and hepatocytes as barriers to systemic therapy for Ad5 using pharmacological interventions [5]. Kupffer cell depletion allowed the virus to escape this barrier by predosing with an irrelevant Ad5 prior to intravenous oncolytic Ad5 injection, but increased the infection and damage of downstream hepatocytes. Detargeting hepatocytes by blocking vitamin K-dependent blood factors including FX binding can be achieved using the drug warfarin [9]. When warfarin was used to inactivate these factors before oncolytic Ad5 injection, this reduced liver infection, but did not improve oncolytic efficacy [5]. Antitumor effects were only improved after i.v. injection when both predosing and warfarin were combined to detarget both Kupffer cells and hepatocytes [5].

An alternate strategy to detarget the liver is to mutate or insert peptides into hexon to block the binding of FX [13,14,15]. To apply this approach for Ad5, we inserted a relatively large 8 kDa biotin acceptor protein (BAP) into the hypervariable region 5 (HVR5) of the hexon surface of oncolytic Ad5 [10,13]. This BAP insertion blocked FX binding, markedly reduced hepatocyte infection [10], consequently reduced liver damage, and increased the maximum tolerated dose of oncolytic Ad5 while maintaining oncolytic efficacy after intravenous injection [13]. 

One can also chemically engineer oncolytic adenovirus with polymers like polyethylene glycol (PEG) or poly-*N*-(2-hydroxypropyl)methacrylamide to “shield” the surface of the virus from interactions with blood proteins and off-target cells [16,17]. PEG is used over the counter as an oral laxative. It is also covalently conjugated to labile therapeutic proteins like interferon or adenosine deaminase to improve their pharmacokinetics. Based on this, a number of groups have covalently PEGylated the surface of replication-defective Ad vectors to improve its pharmacology. PEGylation of Ad protects it from pre-existing neutralizing antibodies to allow multiple administration into immune recipients [18,19,20]. PEGylation blunts innate immune responses against Ad [21,22], reduces the production of new antibody and cellular immune responses against Ad proteins [19]. PEGylation also reduces Ad5 interactions with off target cells, reducing uptake by Kupffer cells [21], endothelial cells, and platelets [23]. PEGylated Ad5 reduces in vitro coxsackie and adenovirus receptor (CAR)-dependent infection by four to five orders of magnitude in vitro, but did not reduce in vivo oncolytic activity of the virus to control tumors derived from LNCaP human prostate cancer cells in nude mice after intravenous injection [17]. This suggested that CAR interactions were less important in vivo for targeting distant tumors after intravenous injection.

These approaches aim to detarget the virus from problematic cells after intravenous injection with particular emphasis on avoiding liver Kupffer cells and hepatocytes. A potentially complementary approach is using conditionally replicating Ads (CRAd) to protect these off-target cells. The predosing, warfarin, BAP, and PEG approaches attempt to physically detarget the virions to prevent any infection and damage of non-cancer cells. In contrast, the CRAd strategy allows these off-target cells to be infected, but aims to prevent the virus from executing its lethal lifecycle in non-cancer cells by controlling early gene E1 or E4 expression with cancer-specific promoters, or by mutating the ability of E1A or E1B proteins to interact with pivotal cellular proteins like pRB, p53, or p300 pathways ([12,24,25,26,27], and reviewed in [7]). 

In this work, we compare physical detargeting by hexon–BAP modification with post-entry targeting using the E1A mutant *dl*1101/07 [28]. We show that Ad oncolytics can generate delays in tumor growth after a single intravenous injection in Ad-permissive Syrian hamsters. Better effects were observed by viruses that were physically detargeted from the liver hepatocytes as compared to viruses whose protective effects on normal cells occur after cell entry. We also test the ability to combine the CRAd strategy with PEG chemical physical detargeting and find that this chemical strategy results in reductions in efficacy following single treatment in hamsters.

## 2. Experimental Section

### 2.1. Cell Lines

HaK hamster kidney cancer cells were obtained from American Type Culture Collection (Manassas, VA, USA). 293 cells were obtained from Microbix (Toronto, ON, Canada). Cells were maintained in Dulbecco’s Minimal Essential Medium with 10% fetal bovine serum (HyClone, Logan, UT, USA).

### 2.2. Viruses

**Figure 1:**
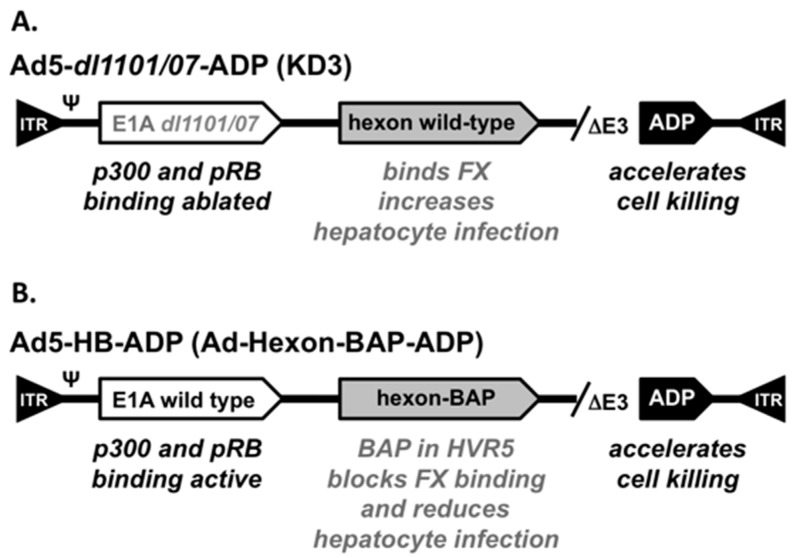
Cartoon of Adenovirus Aerotype 5 (Ad5) Vectors. (**A**) Functional features in Ad5-*dl*1101/07-ADP (see text); (**B**) Functional features in Ad5-HB-ADP (see text).

Wild-type adenovirus serotype 5 (Ad5) was purchased from ATCC. Ad5-ADP has the E3 region deleted before the adenovirus death protein (ADP) [28]. This upstream E3 deletion amplifies ADP expression and accelerates cell killing. Ad5-E1A-*dl*1101/07 was originally generated and described as KD3 [28] (Figure 1A). KD3 was generously provided by Dr. William Wold from Saint Louis University. This virus has two deletions in E1A that are derived from the Ad mutant *dl*1101/07 that ablate the ability of E1A to bind to pRB and p300 rendering the virus relatively cancer-specific [28]. The upstream E3 region of Ad5-E1A-*dl*1101/07-∆E3-ADP is also deleted leaving ADP present. Ad5-hexon-BAP also has the E3 deletion and over-expressed ADP, but has a wild-type E1A protein. For hepatocyte detargeting, this virus displays a 7.4 kDa biotin acceptor peptide (BAP) that was inserted into the HVR5 of hexon [13,29,30]. This BAP blocks FX binding and markedly reduces hepatocyte infection [13]. Adenoviruses were produced from 293 cells in a 10 plate CellStack (Corning Life Sciences, Lowell, MA, USA). Viruses were purified by sequential banding on two CsCl gradients and viral particle numbers were quantitated by OD260. 

### 2.3. Viral PEGylation

Ad5 was PEGylated as in [21]. Following CsCl centrifugation, the preparation was desalted on EconoPac 10-DG columns (BioRad, Hercules, CA, USA) into 0.5 M sucrose in phosphate buffered saline (PBS, 136 mM NaCl, 2.6 mM KCl, 1.7 mM KH_2_PO_4_, 10 mM K_2_HPO_4_). 5 kDa or 20 kDa succinimide-activated PEG (NOF America, White Plains, NY, USA) was reacted with Ad5 at 10 mg/mL PEG for 1 h at room temperature with rotation. Unreacted PEG was removed on a Sephadex G50 (GE Healthcare, Piscataway, NJ, USA) size exclusion column. Mock-treated Ad5 was treated with no addition of PEG.

### 2.4. PEGylation Analysis

Mock PEGylated and PEGylated viruses were analyzed by labeling and sodium dodecyl sulfat poly acrylamide gel electrophoresis (SDS-PAGE) gel separation. 10^10^ vp of each CsCl-banded and desalted virus was separated on 7–15% gradient SDS-PAGE gels (Bio-Rad, Hercules, CA, USA) and total protein was detected by staining with SYPRO^®^ Ruby (Invitrogen, Carlsbad, CA, USA). After succinimide-activated-PEGylation or mock treatment, the viruses were post-reacted with NHS-Oregon Green (Invitrogen) to detect remaining reactive lysines on the virus. Each of these was then separated on SDS-PAGE gels and green fluorescently label capsomers were visualized on a Kodak in vivo F imaging system.

### 2.5. Animal Tumor Models

Animals were housed under the Association for Assessment and Accreditation of Laboratory Animal Care (AALAC) guidelines in the Mayo Clinic Animal Facility. Experiments were performed under animal use protocols approved by the Mayo Clinic Animal Use and Care Committee. All experiments were performed under the provisions of the Animal Welfare Act, Public Health Service Animal Welfare Policy and the principles of the National Institutes of Health Guide for the Care and Use of Laboratory Animals. Four-week old female Syrian golden hamsters (Harlan Sprague Dawley, Indianapolis, IN, USA) were subcutaneously (s.c) injected on the flank with 1 × 10^7^ HaK cells in 100 µL of DMEM. When tumors reached 200 µL in volume, animals were randomized to different groups and were treated a single time by i.v. jugular vein injection of the indicated virus or with PBS. Hamsters were euthanized when the tumor volume exceeded 10% of body weight or if animals were moribund, in distress, or if the skin ruptured over the tumor.

### 2.6. IL-6 Assay

Blood was collected 6 h after infection and was incubated at room temperature for one hour. Serum was separated by centrifuging at 13,000× *g* for 2 min and transferred to a new tube. IL-6 was measured using a BD OptEIA mouse IL-6 enzyme-linked immunosorbant assay (ELISA) set (BD Biosciences, San Diego, CA, USA) according to the manufacturer’s instructions. Briefly, 96 well plates were coated with anti-mouse IL-6 capture antibody at four degrees overnight. The next day, plates were washed 3 times with wash buffer. Serum samples were diluted 1:100 and added to the plate along with serially diluted standard and incubated for 2 h at room temperature. Plates were washed 5 times and working detector was added and incubated for 1 h at room temperature. Plates were washed 7 times and developed with 100 μL substrate solution for 30 min in the dark. 50 μL stop solution was added and OD450 was measured using a Becton Dickinson plate reader with Multimode Detection Software (Becton Dickinson, Franklin Lakes, NJ, USA).

### 2.7. Statistical Analysis

Statistical analysis was performed with Prism (Graphpad, San Diego, CA, USA).

## 3. Results

### 3.1. Comparison of Oncolytic Efficacy between Hepatocyte-Detargeted Ad5 and Replicationally Detargeted CRAd5 after Single Intravenous Injection in Immunocompetent Syrian Hamsters

We previously developed a hepatocyte-detargeted oncolytic Ad5 referred to as Ad-hexon-BAP [13,29,30] (Figure 1B). This virus has wild-type E1A, but most of its E3 region is deleted leaving the adenovirus death protein (ADP) to be over-expressed for accelerated cell killing. Of note for detargeting strategies, Ad-hexon-BAP has a 7.4 kDa biotin acceptor peptide (BAP) inserted into hypervariable region 5 (HVR5) of its hexon protein. This insertion blocks FX binding and markedly reduces hepatocyte infection [10,13]. This modification allows Ad5-hexon-BAP to be used at higher doses than Ad5 by virtue of causing less hepatotoxicity than the unmodified oncolytic. To specify the Ad serotype, the hexon-BAP, ADP features, and for brevity, this virus will be referred to hereafter as Ad5-HB-ADP (Figure 1B).

Ad5-HB-ADP was used as a hepatocyte-detargeted oncolytic in this work. To compare this physically detargeted virus to a replicationally-controlled CRAd detargeting virus, we used KD3 [28] a virus referred hereafter as Ad5-*dl*1101/07-ADP (Figure 1A). This virus has two mutations in E1A (*dl*1101/07) that knock out the ability of E1A to bind cellular pRB and p300 proteins [28]. It has good cancer specificity by virtue of these mutations [28]. This virus has the same E3 deletion and ADP over-expression as Ad5-hexon-BAP [28]. Control viruses included wild-type Ad5 (Ad5-WT) and Ad5-ADP that has a wild-type E1 protein and over-expresses ADP.

Syrian hamsters are thought to be an optimal model to test oncolytic adenoviruses because they are immunocompetent and support adenovirus replication [31]. HaK hamster kidney tumors have been tested extensively in this model [12,31,32,33,34]. Subcutaneous HaK tumors were therefore initiated in hamsters. When tumors reached 200 µL in volume, buffer (PBS) or viruses were injected intravenously a single time with 1.5 × 10^11^ virus particles (vp) by the jugular route to assess the ability of the viruses to treat a distant tumor.

**Figure 2:**
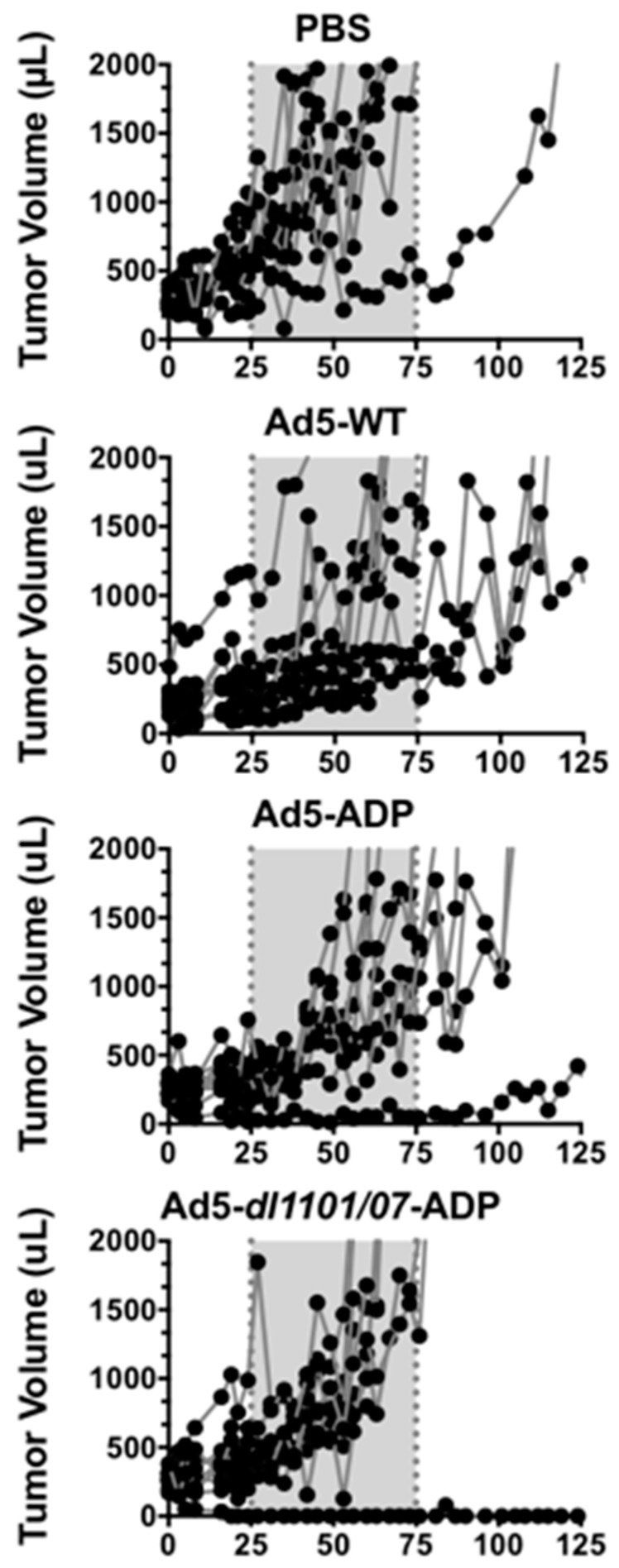

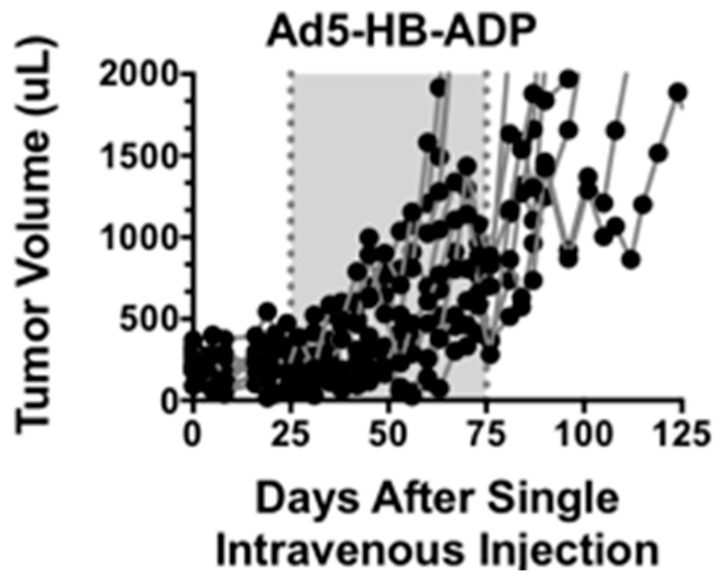
Effect of intravenous adenovirus injection on individual tumor sizes in Syrian hamsters. Hamsters with subcutaneous HaK tumors were injected intravenously a single time with 1.5 × 10^11^ virus particles (vp) of the indicated viruses by the jugular route to assess the ability of the viruses to treat a distant tumor. Tumor sizes in individual animals are shown. The gray box is included to emphasize the increased rapidity in PBS-treated animals when compared to certain Ad-treated animals. This experiment has been repeated once with essentially the same results.

**Figure 3:**
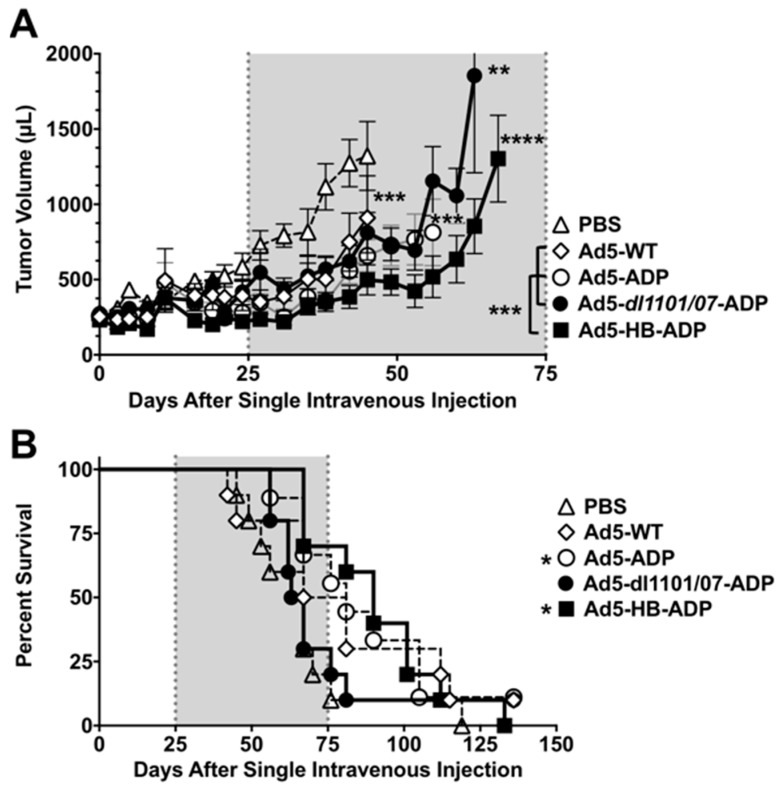
Anticancer activity of Ad5 viruses after intravenous injection. Groups of 9–10 hamsters with subcutaneous HaK tumors were injected a single time with 1.5 × 10^11^ vp of the indicated viruses and tumor sizes were measured. (**A**) Tumor size. Tumor dimensions were measured with calipers and tumor volume was calculated as width^2^ × length × 1/2. The data are shown as mean ± standard error (SE). Tumor size statistics were calculated by a paired repeated measures T test between groups. Asterisks next to lines show significance between the group and the PBS group. Asterisks to the left of the legend with brackets represent the significant difference between Ad5-HB-ADP and other viral groups; (**B**) Effect of a single intravenous injection on survival. An animal lost survival when their tumor exceeded 2000 µL. Kaplan–Meier survival curves were analyzed by Log-rank (Mantel–Cox) test. * *p* < 0.05, ** *p* < 0.01, *** *p* < 0.001, **** *p* < 0.0001.

Tumors in the negative control PBS group grew most rapidly and required the first animal to be euthanized after 45 days (Figure 2, individual tumor sizes and 3A, mean tumor sizes for the group). The line representing mean tumor size terminates at the time of first animal loss in the group, since the group no longer contains all animals that began the study. Most PBS-treated animals exceeded maximum tumor size within 75 days (Figure 2 and Figure 3).

Tumor growth was significantly delayed by Ad5-WT (*p* = 0.007), Ad5-ADP (*p* = 0.0005), and Ad5-HB-ADP groups (*p* = 0.0001) when compared to the PBS group in the (Figure 3A). Most Ad5-*dl*1101/07-ADP treated animals required sacrifice within 75 days, but this was still significantly different to the PBS group (*p* = 0.001). 

Tumor growth was significantly delayed by Ad5-HB-ADP when this group was compared to the other viral groups Ad5-WT, Ad5-ADP, and Ad5-*dl*1101/07-ADP (*p* < 0.001). 

The groups were followed beyond 125 days and Kaplan–Meier survival analysis was performed using tumor size in excess of 2000 µL as an event (Figure 3B). Most animals in the PBS and the Ad5-*dl*1101/07-ADP groups exceeded 2000 µL tumor volumes within 80 days of treatment. Survival was extended for the other groups, however, only Ad5-ADP and Ad5-HB-ADP survival was significantly better than the PBS group (*p* < 0.01, and <0.05, respectively by log-rank analysis). 

While tumor growth delay and survival extension was modest in the virally-treated groups, it should be emphasized that this effect was mediated by a single intravenous injection that required the systemically-distributed virus to reach a distant subcutaneous tumor.

### 3.2. Liver Damage Caused by Intravenous Ad Injection in Hamsters

**Figure 4:**
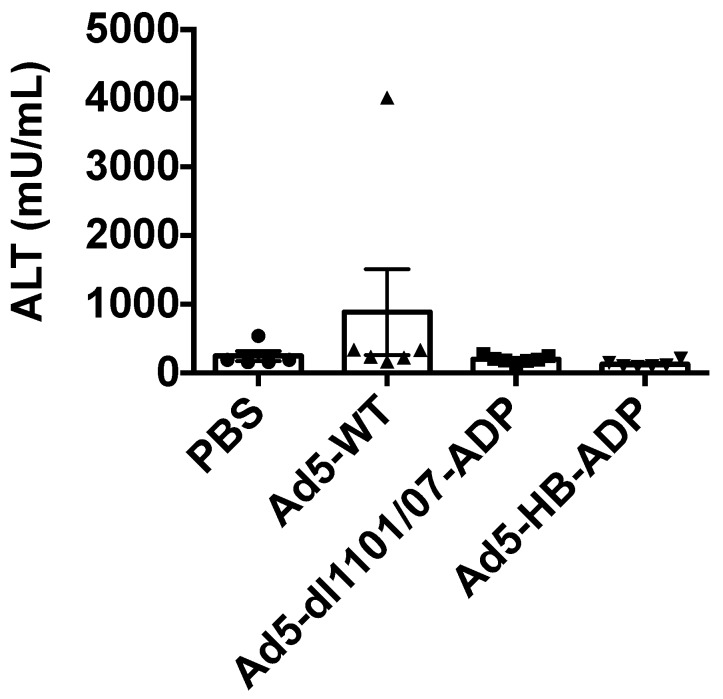
Liver damage after intravenous injection. Syrian hamsters were injected with 5 × 10^11^ vp of the indicated adenoviruses and liver damage was assessed by measurement alanine amino transferase (ALT) release in the blood three days after injection (*n* = 5–7 animals).

We previously showed in mice that Ad5-HB reduced liver damage after intravenous injections in mice by reducing hepatocyte infection [13]. Ad5 with *dl*1101/07 mutations also reduces liver damage after intravenous injection in mice and hamsters [12]. To test this head-to-head in hamsters, animals were injected with the same vectors at the 1.5 × 10^11^ vp dose used in the efficacy study and their blood was tested three days later for release of liver alanine aminotransferase (ALT) release. At these doses, no changes in ALT were observed (data not shown). Hamsters were injected with 7.5 × 10^11^ vp, the highest feasible dose given injection virus concentration and volume parameters. Under these conditions, no significant increases were observed at day three when compared to PBS-treated animals (Figure 4).

### 3.3. Intratumoral Ad5 Treatment in Ad5 Naive and Ad5-Immunized Hamsters

Survival in Figure 3 was defined by tumors that exceeded 2000 µL, which is below the 10% body weight sacrifice criteria in animals that can reach 200 g in mass. Given this, animals that exceeded the 2000 µL tumor volume criteria were treated a second time to explore the effect of Ad5 immunity on tumor control.

**Figure 5:**
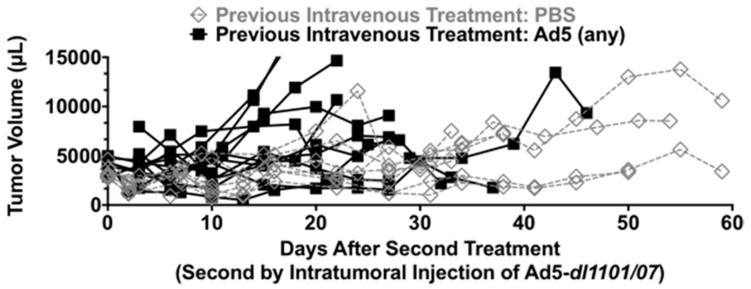
Tumor sizes after second treatment by intratumoral Ad5-*dl*1101/07-ADP injection.

The first Ad treatment was expected to generate robust anti-Ad5 antibodies and T cell responses [31]. This is a concern if trying to readminister by the intravenous route, since high antibody levels would quantitatively neutralize the virus. While this could be problematic, this Ad5 immunity might actually have the utility of amplifying anti-tumor immunity, if Ad5-specific immune cells could be recruited back into the tumor by a second injection [31]. High levels of Ad5 antibodies in the blood would be likely to ablate any ability of the virus to reach the tumor. In contrast, a direct intratumoral injection is not affected by Ad5 antibodies [35]. Therefore to test the effects of potentially beneficial Ad5 immunity on second injection, we tested this by direct intratumoral injection (Figure 5). Given that hexon-BAP virus detargeting only works in the blood, we used Ad5-*dl*1101/07 for these second treatments.

To do this, naive animals (previously with PBS) and Ad5-immune animals (previously treated with any Ad5) were injected intratumorally with Ad5-*dl*1101/07-ADP and tumor sizes were monitored (Figure 5). In this case, there was no obvious additional therapeutic effect of Ad5 administration into the tumors in naive or Ad5-treated animals. For most animals, tumor sizes remained relatively static, with only a few animals’ tumors growing and requiring sacrifice due to this effect. A total of 3/8 (37.5%) of PBS pre-treated animals and 5/12 (41.7%) of Ad5 animals had relatively static tumor sizes and survived after intratumoral Ad5-*dl*1101/07-ADP treatment.

### 3.4. Combining Ad5-dl*1101/07*-ADP CRAd Replication Control with PEG Shielding

**Figure 6:**
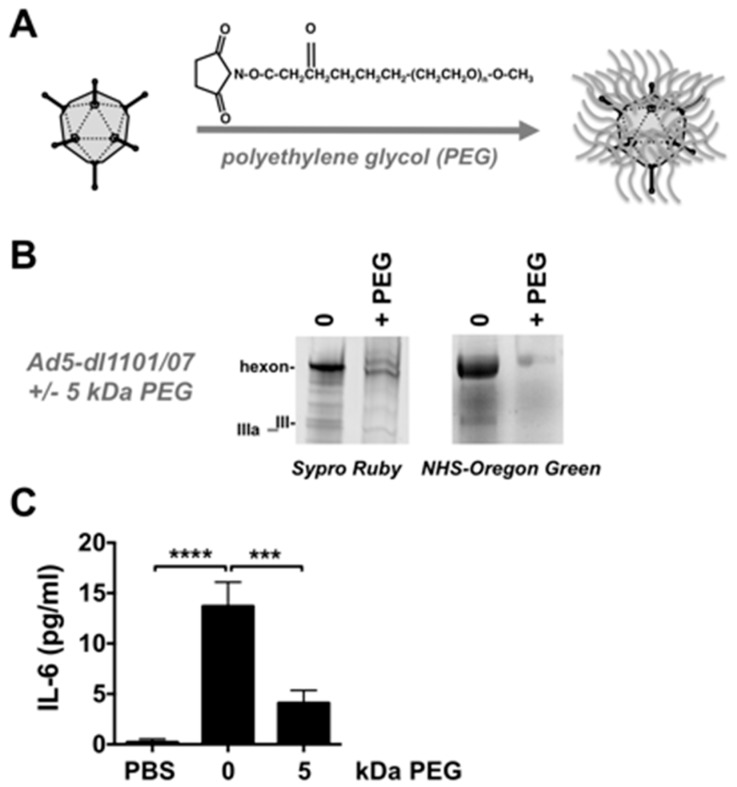
Polyethylene Glycol (PEG) Modification of Ad5-*dl*1101/07-ADP. (**A**) Scheme of chemical shielding with PEG; (**B**) SDS-PAGE analysis of PEGylated Ad5-*dl*1101/07. Viruses were separated on SDS-PAGE gels after desalting for Sypro Ruby staining or were reacted with succinimide-activated NHS-Oregon Green fluorophore by green fluorescence to detect remaining unreacted amines; (**C**) Animals received 3 × 10^10^ vp of virus and were bled 6 h later and sera were tested by IL-6 ELISA. *** = *p* < 0.01. **** = *p* < 0.0001 by analysis of variance (ANOVA).

The physically liver-detargeted Ad5-HB-ADP virus was more effective than the physically untargeted Ad5-*dl*1101/07-ADP CRAd virus, which depends on post-entry targeting effects. To determine if the performance of this physically untargeted virus could be improved, it was chemically shielded with the hydrophilic polymer polyethylene glycol (PEG) (Figure 6A). PEGylation protects Ads from pre-existing neutralizing antibodies [18,19,20] and blunts innate and adaptive immune responses against Ad, including IL-6 responses [19,21,22]. PEGylation also reduces Ad5 uptake by Kupffer cells [21], endothelial cells, and platelets [23]. We previously showed that shielding fully replication-competent Ad5 with PEG preserved oncolytic efficacy in nude mice bearing human LNCaP prostate tumors [17].

To test the combination of PEG and CRAd, Ad5-E1A-*dl*1101/07 was chemically conjugated with lysine-reactive succinimide-activated 5 kDa PEG (Figure 6A). In parallel, we performed a mock PEGylation on control virus that was processed identically with the exception of having no reactive PEG present.

10^10^ vp of unmodified and PEGylated viruses were also separated on SDS-PAGE gels and were stained with SYPRO^®^ Ruby to detect total protein content (Figure 6B). All capsid proteins were stained in proper proportion in the unmodified virus, with the strongest staining of the more abundant hexon. Hexon staining by SYPRO^®^ Ruby was markedly reduced on the 5 kDa PEG virus. This suggested that the 5 kDa PEG was partially shielding hexon from SYPRO^®^ Ruby binding. A second band was also observed above hexon suggesting that PEG conjugation slowed migration. 

Previous work has demonstrated that most NHS-PEG conjugation occurs on lysines on the Ad5 hexon protein [21,36]. To examine if residual unreacted lysines were still present on the viral surfaces, each was reacted with a smaller 0.5 kDa amine-reactive NHS-Oregon Green fluorophore (Figure 6B). SDS-PAGE of 10^10^ vp of the Oregon Green-labeled viruses revealed strong fluorescence labeling of hexon (II) of the unmodified virus, but reduced labeling the PEGylated virus. This suggested that PEG was protecting most of the lysines on Ad5-*dl*1101/07.

### 3.5. Effects of PEG on Innate Immune Responses against Ad5-dl*1101/07* after Intravenous Injection in Mice Bearing Human Tumors

Previous work showed that Ad5 PEGylation can blunt innate immune responses against Ads including IL-6 activation [19,21,22]. Reagents are not available to detect hamster IL-6. Therefore, to test this effect, 3 × 10^10^ vp of unmodified and PEGylated Ad5-*dl*1101/07-ADP viruses were injected intravenously into mice and IL-6 levels in sera were measured 6 h later (Figure 6C). Under these conditions, unmodified Ad5-E1A-*dl*1101/07 produced significant elevations in IL-6 (*p* < 0.0001 by analysis of variance (ANOVA)). In contrast, IL = 6 levels were markedly reduced by the PEGylated vector when compared to Ad5-E1A-*dl*1101/07 (*p* < 0.001).

### 3.6. Oncolytic Efficacy of Unmodifed and PEGylated Ad5-dl*1101/07* after Single Intravenous Treatment of Immunocompetent Hamsters

**Figure 7:**
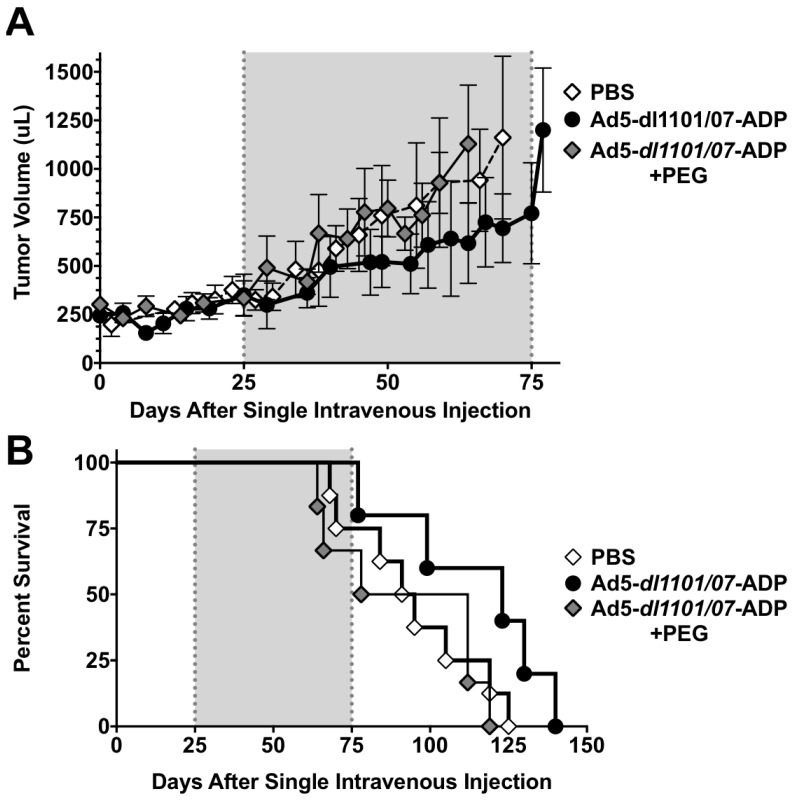
Efficacy of unmodified and PEGylated Ad5-*dl*1101/07 in immunocompetent hamsters. Hamsters with established HaK tumors were injected i.v. by the jugular route with a single dose 3 × 10^10^ vp of Ad5-*dl*1101/07 or Ad5-*dl*1101/07 5 kDa PEG. (**A**) Tumor volumes (*n* = 5 to 8 per group) were measured twice a week and the mean values were calculated from tumor dimensions and all individual tumor size; (**B**) Loss of survival as described in Figure 3.

To test the Ad5-*dl*1101/07-ADP CRAd with and without PEGylation, the viruses were injected a single time by the intravenous jugular route with PBS or with 1.5 × 10^11^ vp dose (Figure 7). Under these conditions, hamsters treated with a single low intravenous dose of Ad5-*dl*1101/07 grew slightly slower than PBS controls (Figure 7A). Tumor growth in the 5 kDa PEGylated Ad5-*dl*1101/07 group was virtually identical to the PBS group. Kaplan–Meier survival analysis recapitulated tumor growth in the groups with the PBS and 5 kDa PEG group being similar (Figure 7B). Unmodified Ad5-*dl*1101/07 mediated better survival than the other groups (*p* = 0.08) although this did not quite meet the *p* < 0.05 threshold of significance. However, the median survival of 97 days for the PBS group was extended to 123 days by only one intravenous injection of Ad5-*dl*1101/07 treatment. Median survival for the 5 kDa PEG group was 95 days.

## 4. Discussion

This work compared and combined technologies to detarget oncolytic adenoviruses for systemic therapy of cancer. For these DNA viruses, there are several approaches that can be adopted to increase cancer-specificity before or after cell entry occurs [2].

We compared the physical detargeting of Ad5 away from hepatocytes by modification of the virus’ hexon protein with post-entry replication-detargeting with a CRAd virus mutated for reduced p300 and pRB interactions. 

Previous work with the physically detargeted replication-defective or replication-competent Ad5-hexon-BAP viruses demonstrated that this hexon modification reduces hepatocyte infection after intravenous injection and allows 10-fold higher doses to be used without hepatotoxicity in mice [10,13]. Similarly, the Ad5 CRAd Ad5-*dl*1101/07-ADP was shown previously to reduce liver damage 10-fold after intravenous injection [12]. When we directly compared the hepatocyte-detargeted Ad5-HB-ADP to the KD3 CRAd Ad5-*dl*1101/07-ADP in immunocompetent hamsters, both mediated significant delays in tumor growth. However, the hexon-modified virus had better efficacy than the CRAd after single intravenous injection. This could be due to less virus being sequestered in the liver after intravenous injection and/or could be due to slight loss in oncolytic efficacy that is reported when *dl*1101/07 mutations are introduced into E1 [12]. Retreatment of the animals by direct intratumoral injection did not appear to improve tumor control. No benefit or cost was obvious in the animals that had previously been exposed to Ad or not. However, these tumors were fairly large at the time of intratumoral injection, so therapeutic effects might not be able to be appreciated. 

Given the efficacy of the *dl*1101/07 CRAd, we tested whether it could be combined with an alternate detargeting strategy, wherein the surface of Ad is shielded with the hydrophilic polymer PEG. Previous work has shown that Ad5 PEGylation blunts, but does not ablate, virtually every immunologic or interaction phenomena associated with intravenous Ad5 injection. PEGylation of Ad5 partially protects it from neutralizing antibodies [18,19,20]. PEGylation reduces Ad5 interactions with non-target cells including Kupffer cells [21], endothelial cells, red blood cells, and platelets [23]. PEGylation also blunts innate immune responses [21,22]. Therefore, it seemed that PEG might partner well with the CRAd’s ability to preserve non-tumor tissues from viral lysis. In this study, we confirmed that PEGylation can blunt IL-6 responses against the CRAd Ad5-*dl*1101/07.

Previous work PEGylating E1-intact Ad5 demonstrated that this modification retained oncolytic efficacy after intravenous treatment of LNCaP tumors in immunodeficient mice [17]. In this previous work, we injected the Ads in two doses separated by 4 h to “predose” the animals wherein the first bolus of Ad5 eliminates Kupffer cells, so the second virus is not destroyed by these macrophages [5]. Here we did not predose, but used only one injection of Ad5. Therefore, much of the unmodified virus is likely neutralized by liver Kupffer cells, but PEG should reduce this effect [21]. This study is also different, since we applied a CRAd rather than a fully replication-competent Ad5 and they were tested in larger and immunocompetent Syrian hamsters rather than immunodeficient mice. When tested here in immunocompetent hamsters, a single intravenous dose of unmodified Ad5-*dl*1101/07 mediated modest, but detectable delays in tumor growth. In contrast, Ad5-*dl*1101/07 modified with 5 kDa PEG was no better than the PBS in the immunocompetent animals. This is consistent with recent work showing PEGylation of oncolytic Ad6 can also reduce efficacy after intravenous injection in difficult to cure tumor models [37]. Previous work demonstrated that 5 kDa PEG actually increases the uptake and transduction of liver hepatocytes by Ad5 [17,21,38], so the lack of efficacy after a single intravenous injection may be due to increased loss of virus to the parenchyma of the liver. 

Previous work has shown strong therapeutic effects after intravenous treatments in immunodeficient mice bearing human tumors [17,21,37,38]. In contrast, the therapeutic effects were modest in the hamsters in previous work and in this study. Single intravenous injection was performed in the HaK hamster model to provide a single dose benchmark for future more complex dosing and combinations. It should also be noted that previous efficacy testing in this model has usually performed injected Ad5 directly into tumors and not by the systemic intravenous route [12,31,32,33]. While HaK tumors do support species C adenovirus replication as demonstrated for Ad5 and Ad6 [34], viral replication in hamster cells is reduced approximately four-fold relative to replication in human cells [39]. 

It should be emphasized that are several overlapping mechanisms that sequester Ad5 in the liver and detargeting one or two may have only partial effects. Physical retargeting via hexon-BAP and PEG can change physical sequestration, but modifying Ad activation with E1 mutations will not affect sequestration at all. In mice, 98% of intravenous Ad5 is absorbed by the liver [4]. Approximately 65% of the liver cells are hepatocytes, 25% are liver sinusoidal endothelial cells (LSECs), and 7% are Kupffer and other cells [40]. Previous work by Shayakhmetov’s group showed that one must detarget Kupffer cells, LSECs, and hepatocytes to significantly reduce all Ad5 sequestration by the liver [41]. In this work, the detargeting strategies each combat only one or two of these overlapping sequestration mechanisms. 

Kupffer cells are the resident macrophage in the liver [6] and are thought to absorb most injected Ad5 [4]. Of the detargeting strategies tested, only PEGylation is thought to detarget Kupffer cells [21]. LSECs are another mechanism for Ad sequestration from the blood [41]. There are approximately four times as many LSECs as Kupffer cells, so even less efficient uptake by these cells may significantly alter the fate of systemically administered Ads. Of the detargeting strategies tested, PEGylation appears able to reduce endothelial cell uptake in vitro [23], but it is unclear if this occurs in vivo.

Both Kupffer and LSECs reside in the blood supply feeding the liver parenchyma. Therefore, intravenous Ad5 must evade both Kupffer cells and LSECs before reaching hepatocytes within the parenchyma. In the presence of FX, Ad5 is protected from complement and partially protected from destruction by Kupffer cells, this is mediated by complement fixation [42,43]. BAP insertion blocked FX-binding to markedly reduce hepatocyte infection and sequestration [10]. The BAP reduces but overall uptake of Ad5 in the whole liver in mice with intact natural antibody and complement levels, but not in nude or *Rag−/−* mice [13]. It is unclear if the presence of the large BAP on hexon reduces natural antibody or complement deposition on Ad and Kupffer cell sequestration.

While tumor efficacy was modest, it should be emphasized that these therapeutic effects were mediated by a single intravenous injection in these larger immunocompetent animals. Under these conditions, the viruses suffer a sequestration and inactivation in the blood and must “find” the distant subcutaneous tumors to have efficacy. Given this, these data suggest that oncolytic Ads can have utility in systemic therapeutics, particularly if they are engineered to avoid sequestration by liver cells.
